# Supplementary material for: Later Growth Cessation and Increased Freezing Tolerance Potentially Result in Later Dormancy in Evergreen Iris Compared with Deciduous Iris
Source: Int J Mol Sci. 2022 Sep 22;23(19):11123. doi: 10.3390/ijms231911123 (PMC9569662; doi:10.3390/ijms231911123)
Supplement: Supplementary file 1 [file ijms-23-11123-s001.zip › ijms-1908891-supplementary.pdf]

Supplemental Table S1

Genes and corresponding primers used for quantitative real-time PCR.

Gene name	Gene full name	*Arabidopsis homolog* ID	Functional description	Forward/reverse primer sequence (5'-3')
Reference gene				
Actin	*Actin*	AT5G09810	cell division,cytoskeleton organization	ACATTGTGCTCAGTGGAGGT/ ACTTCCTCTCTGGTGGAGCA
Marker genes				
CYCD1;1	*cyclin D1;1*	AT1G70210	mitotic cell cycle phase transition	TGGAGGGTGCAAGGTTCATC/ AGAGTGGGTTCCGCTAGGAT
CDKB2;2	*cyclin-dependent kinase B2;2*	AT1G20930	regulation of mitotic cell cycle	GAAGCCCCACAACCTTCTGA/ GTCGAGTAATGAGTCGCCCC
SVP2	*short vegetative phase 2*	AT2G22540	positive regulation of dormancy	AATCATTGAAAAGCGCGGCA/ AGTTGCTCAGTGCTCTCAGTT
FLC1	*flowering locus c 1*	AT1G69120	floral meristem determinacy, flower development, maintenance of floral meristen identity	CGATGTCGGGCTCATCATCTT/ TGAAAATTCCCGGTATCGGCT
FLC2	*flowering locus c 2*	AT1G69120	floral meristem determinacy, flower development, maintenance of floral meristen identity	ATGATTAGCAAGCTCCGCGA/ CCCTCAGGTGGATTAGGTTGG
FUL2	*fruitfull*	AT5G60910	positive regulation of flower development	CTTGAGAGAAAGGAGGGGGC/ GACGGCGATGAGAACTCTGT
Genes associated with phytohormone biosynthesis, catabolism and signal transduction				
NCED1	*9-cis-epoxycarotenoid*	AT3G63520	abscisic acid biosynthetic process	AGAAAGCACGGTTTGGTGTG/ CACCCTCCTCCCAAGCATTT
ABA2	*ABA deficient 2*	AT1G52340	abscisic acid biosynthetic process	GACGAACGAGGAGTTCGAGG/ TCCACCACCAAGTTGTGTCC
AAO3	*abscisic aldehyde oxidase 3*	AT2G27150	abscisic acid biosynthetic process	TCCTGACCACAATGTCCGTG/ ATTCTAACAGGTCGCCGCAA
CYP707A1	*cytochrome P450, family 707,*	AT4G19230	abscisic acid metabolic process	TTTCGCTCTACCCACGGATG/ GGAGGCCAACTGCGTATTCT
PYL8	*PYR1-LIKE 8*	AT5G53160	positive regulation of abscisic acid-activated signaling pathway	ATGGTGATGAACGGACACGG/ CTCACGAACGGCTTGTACCT
PP2C5	*phosphatase 2C5*	AT2G40180	abscisic acid-activated signaling pathway	ACGAGCGGGAAAGAATCGAA/ GATCCGGTATCACCCACTGC
SnRK1	*snf1-related protein kinase 1*	AT3G01090	positive regulation of abscisic acid-activated signaling pathway, starch metabolic process	GTTCCACTGGGCATCCTGAA/ TCCTCCCTCGTCTTGTAAAGC
SnRK2.6	*snf1-related protein kinase 2.6*	AT4G33950	abscisic acid-activated signaling pathway, sucrose metabolic process	ACTTCTTGACGGAAGCCCAG/ TGCAACCTTGCCATCGTACT
ABI8	*ABA insensitive 8*	AT3G08550	response to abscisic acid	TTGTTATGGCACGGGATGCT/ ACGTCATCACGCTCAATGCT
BZIP12	*ATBZIP12*	AT2G41070	abscisic acid-activated signaling pathway	GGAAAGGATCGAGACAGCGT/ GGCCTGAGCCATTACCCATT
ABF1	*abscisic acid responsive element-binding*	AT1G49720	abscisic acid-activated signaling pathway	TTAGAGGAGGGTTCGGTCGT/ CCCGTCGAGCTGAGGATAAC
RGA	*repressor of GA*	AT2G01570	negative regulation of gibberellic acid mediated signaling pathway, response to abscisic acid	CCTCTACTGTTACGACCGCC/ GGCTTCTTGGGAGTCAACGA
GAI	*gibberellic acid insensitive*	AT1G14920	negative regulation of gibberellic acid mediated signaling pathway, response to abscisic acid	CTCGACGTTCTCCGACATCC/ AAGTCGATCACGTGGACCTT
PIF3	*phytochrome interacting factors 3*	AT1G09530	gibberellic acid mediated signaling pathway	TGCATGTCGCCTATGATGCT/ ATGTCGAGCATCCCCATTCC
GA20ox2	*gibberellin 20 oxidase 2*	AT5G51810	gibberellin biosynthetic process	TCATTCAAACCCTCGGCGAA/ GAGTCGTTGCCCTCGAAGAA
GA2ox2	*gibberellin 2-oxidase 2*	AT1G30040	gibberellin catabolic process	GAGGCCGCTAGGTTCTTCTC/ TGCGTCTTGCTGGATGTGAT
SPY	*spindly*	AT3G11540	gibberellic acid mediated signaling pathway	AGGGATGATGGCTTGCAGAC/ CAGGCAGACGAACCAACTCT
EXPA1	*expansin 1*	AT1G69530	response to gibberellin	ATCACCAATGTAGGCGGAGC/ CCTGGCCGTTGAGATAGGTG
Genes associated with carbohydrate metabolism				
UPG2	*UDP-glucose pyrophosphorylase 2*	AT5G17310	UDP-glucose metabolic process	AAGGCTGTAAAGCGCCTTGT/ CAAAGAAGCGTATTGCGGCT
APL2	*AGPase large subunit 2*	AT1G27680	starch biosynthetic process	CTAAGGCTGTCGCACCAAGA/ TCGGAGCCAAAGTCGTTTGA
APS1	*ADP-glucose pyrophosphorylase small subunit 1*	AT5G48300	starch biosynthetic process	TGCAACAGCTTTCGGTCTGA/ AGGGCATCTCTTTTGCCCTT
GBSS1	*granule bound starch synthase 1*	AT1G32900	starch biosynthetic process	ACCGAGTCTTGACAGTGAGC/ TCGTTTGCTGGATCCCACTC
SS2	*starch synthase 2*	AT3G01180	starch biosynthetic process	CACGGGAGCTCAAGACATCA/ TGGCGTAACCATCGGACTTC
SBE2.2	*starch branching enzyme 2.2*	AT5G03650	starch biosynthetic process	GGATTGGAAGATGGGCGACA/ TGACGGTCTATCCAGAGCCA
ISA1	*isoamylase 1*	AT2G39930	starch biosynthetic process	CCAGCCTATGGGATGCTGTT/ AACTTGGTAAAGGCCCCCAG
PUL1	*pullulanase 1*	AT5G04360	starch biosynthetic process	ATATACGGGGAAGGCTGGGA/ AATGGTGAGCCGCCTAGAAC
SPS3	*sucrose phosphate synthase 3F*	AT1G04920	sucrose biosynthetic process	GCCATCAGGTCGGACATTCA/ GTGCCCGGGTAATACACCTC
SUS1	*sucrose synthase 1*	AT5G20830	sucrose metabolic process	CCTACATTTGCGACGGCCTA/ TCGCTTCAATCCTCCTGACG
SUS3	*sucrose synthase 3*	AT4G02280	sucrose metabolic process	AAGCTGGGCTGTCTCGAATC/ TCTCGCGTCTTTCGAGCTTT
SUS4	*sucrose synthase 4*	AT3G43190	sucrose metabolic process	ATGCTGTGGGAACCACTTGT/ GCCACACATCGAAACGTGAG
CINV2	*cytosolic invertase 2*	AT4G09510	sucrose catabolic process	AAGCGTTTGCATGCCTTGAG/ CCACCACGGGTAGGCATAAA
CWINV1	*cell wall invertase 1*	AT3G13790	sucrose metabolic process	GGCAGATGTGGTGGTGTCAT/ CCACCCTTGGTTCCCTTCTT
VIN1	*vacuolar invertase 1*	AT1G62660	sucrose metabolic process	GCGGGAGACTGATATTGCCA/ TGGTGATGCCGCTGAAATCT
VIN2	*vacuolar invertase 2*	AT1G12240	sucrose metabolic process	CATCACCATCGACACGGGAT/ TTGTAACCGACATCCGCCTC
HKL1	*hexokinase-like 1*	AT1G50460	glucose 6-phosphate metabolic process, hexose metabolic process	TAGCTGCTGCCGGTATTGTAG/ TAAAGGCCTCCCTCAATGGC
SUT4	*sucrose transporter 4*	AT1G09960	sucrose metabolic process, sucrose transport	TTCGACACTGATTGGGTGGG/ GACCAAGCCTGATCCCCATT
AMY3	*alpha-amylase-like 3*	AT1G69830	starch catabolic process	AGAGCTGAAAGCCCTTGTGAA/CCCA GTTTAAGCGTCCACCA
BAM1	*beta-amylase 1*	AT3G23920	starch catabolic process	AGCAAGACGGTGTCTGGAAG/ TGTTATACTGGCCTGCGTCG
BAM3	*beta-amylase 3*	AT4G17090	starch catabolic process	GGAAAGAACGGACCACACGA/ GGAGCTTGCTCGAGTACCAG
DPE2	*disproportionating enzyme 2*	AT2G40840	starch catabolic process	AGACACTGCCTGTCCTGTTG/ CCTCGCTTGGCATTCTCTGA
GWD3	*ATGWD3*	AT5G26570	starch catabolic process	CTACAGAGGCCATGCTTGCT/ TCGGTCAAGCTTCCTGCATT
LSF1	*LIKE SEX4 1*	AT3G01510	starch catabolic process	CAACCACCTGCTTGGAATGC/ CCAATTGACACGCTCGCTTT
MEX1	*maltose excess 1*	AT5G17520	starch catabolic process	GTTGGCTCGGGTCTTGTTCT/ AACGTCGACCACATCACCTG
SEX4	*starch-excess 4*	AT3G52180	starch catabolic process	GTAAACGGCCATGCTTTCCG/ TTCTCTGCCCCCATCCAATG
TOR	*target of rapamycin*	AT1G50030	sucrose mediated signaling, positive, regulation of abscisic acid biosynthetic process	TGGTTGGGTGCCTAACTGTG/ TGTTCTCCAAAGCATGCCCA

Supplementary Table S2

Correlation analysis of gene expression levels in IT during artificial dormancy induction.

	ABA2	*AMY3*	*APL2*	*APS1*	*BAM1*	*BAM3*	*CINV2*	*CWINV1*	*DPE2*	*GBSS1*	*GWD3*	*HKL1*	*ISA1*	*LSF1*	*MEX1*	*PUL1*	*SBE2.2*
ABA2	1																
AMY3	-0.42	1															
APL2	-0.461	-0.219	1														
APS1	0.253	0.155	-0.567	1													
BAM1	-0.595	0.881**	-0.076	-0.097	1												
BAM3	-0.632	0.865**	0.281	-0.321	0.915**	1											
CINV2	-0.022	0.509	-0.576	0.827**	0.377	0.076	1										
CWINV1	0.068	0.452	0.107	-0.553	0.477	0.588	-0.256	1									
DPE2	0.760*	-0.670*	-0.271	0.364	-0.706*	-0.806*	0.04	-0.238	1								
GBSS1	-0.093	-0.184	0.351	-0.785*	0.048	0.221	-0.713*	0.621	0.296	1							
GWD3	0.569	-0.663*	-0.188	-0.358	-0.595*	-0.548	-0.42	0.008	0.656	0.211	1						
HKL1	0.754	-0.276	0.119	-0.497	-0.135	-0.057	-0.258	0.609	0.7	0.525	0.627	1					
ISA1	-0.608	0.875**	-0.616	0.616	0.672*	0.558	0.855**	-0.119	-0.593	-0.687	-0.66	-0.635	1				
LSF1	-0.715*	0.207	0.52	-0.285	0.416	0.512	-0.204	-0.035	-0.518	0.534	-0.538	-0.281	-0.09	1			
MEX1	0.583	-0.511	0.023	-0.488	-0.337	-0.195	-0.438	0.312	0.634	0.502	0.807**	0.990**	-0.64	-0.349	1		
PUL1	0.089	0.064	-0.45	0.939**	-0.305	-0.446	0.696*	-0.486	0.248	-0.764*	-0.071	-0.453	0.47	-0.562	-0.448	1	
SBE2.2	0.525	0.195	-0.573	.747*	-0.26	-0.372	0.607	-0.049	0.404	-0.593	0.21	-0.002	0.29	-0.843**	-0.046	0.852**	1
SEX4	0.637	-0.035	-0.361	-0.27	0.011	-0.004	-0.062	0.638	0.404	0.362	0.606	0.801*	-0.057	-0.456	0.812*	-0.336	0.185
SnRK1	-0.428	0.841**	-0.19	0.558	0.589	0.49	0.703*	-0.16	-0.607	-0.594	-0.805**	-0.653	0.977**	0.101	-0.779*	0.532	0.474
SNRK2.6	-0.617	0.62	-0.248	0.323	0.690*	0.604	0.532	-0.349	-0.57	-0.516	-0.750*	-0.809*	0.753*	0.513	-0.663	-0.037	-0.304
SPS3	-0.37	0.936**	-0.498	0.62	0.690*	0.62	0.941**	0.193	-0.459	-0.647	-0.49	-0.331	0.922**	-0.221	-0.516	0.517	0.456
SS2	-0.833*	0.772**	-0.241	0.357	0.752*	0.703*	0.48	0.133	-0.649	-0.357	-0.673*	-0.555	0.821**	0.233	-0.592	0.141	-0.142
SUS1	-0.162	0.454	-0.52	0.927**	0.052	-0.09	0.951**	-0.24	-0.128	-0.62	-0.345	-0.437	0.611	-0.54	-0.466	0.750*	0.584
SUS3	-0.599	0.856**	-0.139	-0.197	0.947**	0.901**	0.288	0.492	-0.726*	-0.022	-0.497	-0.182	0.721*	0.298	-0.238	-0.249	-0.143
SUS4	0.202	-0.706*	0.143	0.172	-0.737**	-0.778**	-0.208	-0.767*	0.302	-0.322	0.181	-0.373	-0.415	-0.019	-0.263	0.278	-0.07
SUT4	-0.698*	0.655*	0.036	-0.039	0.744**	0.722*	0.244	0.137	-0.734*	-0.348	-0.588	-0.666	0.68	0.508	-0.554	-0.039	-0.269
TOR	0.394	0.013	-0.577	0.976**	-0.23	-0.418	0.727*	-0.627	0.403	-0.758*	-0.281	-0.566	0.503	-0.262	-0.426	0.978**	0.770*
UGP2	-0.777*	0.42	0.454	-0.276	0.591	.771*	-0.091	-0.063	-0.854**	-0.132	-0.649*	-0.587	0.362	0.671*	-0.488	-0.39	-0.624
VIN1	0.185	-0.709*	0	0.292	-0.769**	-0.776*	-0.359	-0.842**	0.372	-0.329	0.376	-0.397	-0.286	-0.159	-0.191	0.362	0.011
VIN2	-0.65	0.755**	0.016	-0.245	0.930**	0.876**	0.212	0.489	-0.709*	0.174	-0.453	-0.105	0.583	0.444	-0.25	-0.34	-0.343
AAO3	-0.03	0.421	-0.114	-0.309	0.543	0.57	-0.014	0.878**	-0.047	0.432	-0.027	0.807*	0.007	-0.117	0.424	-0.517	-0.069
ABF1	-0.086	0.643	-0.621	0.773*	0.335	0.236	0.919**	-0.108	-0.395	-0.811*	-0.425	-0.38	0.811*	-0.468	-0.48	0.719*	0.710*
ABI8	0.437	0.289	-0.690*	0.339	0.048	-0.04	0.496	0.203	0.335	-0.309	0.331	0.211	0.441	-0.694*	0.32	0.492	0.823**
BZIP12	-0.372	0.893**	-0.326	0.057	0.866**	0.824**	0.494	0.544	-0.506	-0.15	-0.387	-0.009	0.771*	0.021	-0.193	0.03	0.204
CYP707A1	0.579	-0.148	-0.129	-0.28	-0.093	-0.054	-0.101	0.531	0.58	0.366	0.536	0.931**	-0.338	-0.473	0.887**	-0.188	0.272
GAI	0.458	0.041	-0.781*	0.369	-0.171	-0.34	0.473	-0.296	0.265	-0.596	0.507	-0.035	0.458	-0.762*	0.224	0.676	0.848**
NCED1	0.635	0.128	-0.555	0	0.066	0.022	0.24	0.628	0.355	0.017	0.509	0.689*	0.029	-0.611*	0.602	-0.047	0.516
PP2C5	0.738	-0.835**	0.152	-0.251	-0.779**	-0.665	-0.533	0.06	0.706	0.355	0.869**	0.738*	-0.903**	-0.516	0.833*	-0.072	0.087
PYL8	-0.572	0.701*	-0.016	-0.334	0.864**	0.948**	0.375	0.587	-0.546	0.11	-0.187	0.391	0.576	0.193	0.099	-0.419	-0.238
RGA	-0.474	0.654*	0.278	-0.524	0.848**	0.856**	0.043	0.742*	-0.723*	0.245	-0.226	0.339	0.323	0.288	0.142	-0.582	-0.323
EXPA1	0.538	-0.479	0.223	-0.413	-0.361	-0.275	-0.412	0.442	0.656	0.562	0.745**	0.952**	-0.684*	-0.331	0.956**	-0.289	0.026
GA20ox2	0.147	-0.1	-0.36	0.927**	-0.249	-0.445	0.534	-0.622	0.466	-0.345	-0.358	-0.539	0.221	-0.026	-0.505	0.963**	0.227
GA2ox2	0.800*	-0.404	0.072	-0.197	-0.466	-0.398	-0.244	0.438	0.519	0.151	0.699*	0.816*	-0.68	-0.631	0.928**	0.021	0.616
PIF3	0.551	-0.447	0.012	-0.446	-0.311	-0.223	-0.404	0.483	0.537	0.387	0.794**	0.956**	-0.571	-0.534	0.954**	-0.353	0.093
SPY	0.857**	-0.044	-0.704*	0.308	-0.235	-0.197	0.261	0.271	0.525	-0.313	0.313	0.671	-0.194	-0.653*	0.425	0.101	0.619
CYCD1;1	0.109	-0.865**	0.644	-0.129	-0.785**	-0.773*	-0.576	-0.459	0.577	0.199	0.27	0.202	-.765*	-0.027	0.257	0.021	-0.223
CDKB2;2	0.094	-0.852**	0.27	0.126	-0.829**	-0.855**	-0.211	-0.625	0.6	0.006	0.148	0.032	-0.685	-0.013	0.132	0.038	-0.32
SVP2	-0.203	0.751**	-0.395	0.730*	0.617*	0.378	0.822**	-0.029	-0.198	-0.386	-0.754**	-0.436	0.915**	0.052	-0.675	0.423	0.372
FUL2	-0.791*	0.657*	0.094	-0.291	0.920**	.903**	0.312	0.293	-0.735	0.141	-0.57	-0.194	0.499	0.629	-0.318	-0.423	-0.571
FLC1	-0.1	-0.591	0.511	-0.443	-0.431	-0.256	-0.662*	-0.477	-0.123	0.102	0.239	-0.165	-0.541	0.326	0.228	-0.286	-0.518
FLC2	-0.426	-0.464	0.554	-0.415	-0.204	-0.124	-0.569	-0.482	-0.402	0.218	-0.085	-0.366	-0.357	0.678*	-0.22	-0.314	-0.689*

	SEX4	*SnRK1*	*SNRK2.6*	*SPS3*	*SS2*	*SUS1*	*SUS3*	*SUS4*	*SUT4*	*TOR*	*UGP2*	*VIN1*	*VIN2*	*AAO3*
SEX4	1													
SnRK1	-0.414	1												
SNRK2.6	-0.424	0.662	1											
SPS3	-0.093	.931**	0.553	1										
SS2	-0.373	.793*	0.779*	0.724*	1									
SUS1	-0.143	.786*	0.098	0.596	0.507	1								
SUS3	0.155	0.648	0.656*	0.748*	0.809**	0.077	1							
SUS4	-0.466	-0.269	-0.173	-0.576	-0.461	-0.084	-0.768**	1						
SUT4	-0.538	.789*	0.772*	0.586	0.888**	0.264	0.863**	-0.328	1					
TOR	-0.344	0.451	0.32	0.457	0.15	0.788*	-0.284	0.266	-0.167	1				
UGP2	-0.514	0.607	0.732*	0.169	0.532	-0.192	0.617	-0.148	0.785*	-0.262	1			
VIN1	-0.55	-0.239	-0.171	-0.524	-0.336	0.146	-0.735*	0.937**	-0.326	0.438	-0.198	1		
VIN2	-0.097	0.499	0.659*	0.606	0.776**	-0.052	0.976**	-0.702*	0.866**	-0.358	0.676*	-0.709*	1	
AAO3	0.391	-0.11	-0.104	0.265	0.328	-0.104	0.547	-0.790**	0.307	-0.441	-0.045	-0.754*	0.581	1
ABF1	-0.052	.843**	0.398	0.922**	0.506	0.725*	0.446	-0.331	0.471	0.681*	0.214	-0.226	0.274	0.078
ABI8	0.607	0.226	-0.097	0.514	-0.023	0.412	0.212	-0.458	-0.19	0.377	-0.562	-0.359	-0.029	0.257
BZIP12	0.13	.723*	0.5	0.888**	0.746*	0.336	0.922**	-0.844**	0.680*	-0.083	0.328	-0.772**	0.845**	0.638*
CYP707A1	0.655*	-0.456	-0.576	-0.069	-0.302	-0.202	-0.029	-0.452	-0.319	-0.304	-0.602	-0.399	-0.051	0.680*
GAI	0.498	0.081	0.017	0.393	-0.203	0.654	0.037	-0.032	-0.243	0.498	-0.463	0.007	-0.226	-0.145
NCED1	0.843**	-0.265	-0.274	0.222	-0.252	-0.04	0.232	-0.534	-0.281	0.036	-0.545	-0.612	0.039	0.527
PP2C5	0.256	-.890**	-0.921**	-0.760*	-0.784*	-0.308	-0.740*	0.386	-0.712*	-0.219	-0.684*	0.391	-0.670*	0.097
PYL8	0.066	0.107	0.44	0.713*	0.606	-0.144	0.927**	-0.773**	0.744*	-0.457	0.454	-0.808*	0.937**	0.750*
RGA	0.178	0.131	0.319	0.464	0.446	-0.314	0.881**	-0.815**	0.663*	-0.615	0.64	-0.872**	0.882**	0.778**
EXPA1	0.638	-0.745*	-0.780**	-0.44	-0.529	-0.392	-0.34	-0.164	-0.554	-0.41	-0.613	-0.166	-0.277	0.485
GA20ox2	-0.459	0.369	0.243	0.096	0.326	0.755*	-0.401	0.33	-0.163	0.953**	-0.253	0.559	-0.354	-0.441
GA2ox2	0.563	-0.651	-.820**	-0.318	-0.671	-0.21	-0.411	0.04	-0.539	-0.219	-0.680*	-0.05	-0.417	0.367
PIF3	0.806**	-0.696*	-0.664	-0.396	-0.497	-0.366	-0.167	-0.277	-0.48	-0.438	-0.548	-0.241	-0.199	0.476
SPY	0.676	-0.132	-0.342	0.047	-0.44	0.078	-0.167	-0.149	-0.379	0.369	-0.625	-0.263	-0.283	0.289
CYCD1;1	-0.224	-0.587	-0.569	-0.813**	-0.535	-0.279	-0.856**	0.746*	-0.524	-0.007	-0.25	0.724*	-0.693*	-0.422
CDKB2;2	-0.212	-0.458	-0.376	-0.779*	-0.548	-0.234	-0.922**	0.818**	-0.575	0.174	-0.353	0.782*	-0.781**	-0.548
SVP2	-0.124	0.869**	0.643	0.912**	0.868**	0.757*	0.54	-0.404	0.455	0.63	0.119	-0.466	0.463	0.077
FUL2	-0.134	0.24	.756*	0.437	0.677*	-0.177	0.833**	-0.508	0.699*	-0.467	0.667	-0.596	0.884**	0.343
FLC1	-0.295	-0.476	-0.131	-0.770*	-0.436	-0.49	-0.429	0.715*	-0.1	-0.327	0.34	0.704*	-0.333	-0.515
FLC2	-0.564	-0.292	0.094	-0.574	-0.106	-0.421	-0.253	0.695*	0.179	-0.361	0.625	0.645	-0.087	-0.485

	ABF1	*ABI8*	*BZIP12*	*CYP707A1*	*GAI*	*NCED1*	*PP2C5*	*PYL8*	*RGA*	*EXPA1*	*GA20ox2*	*GA2ox2*	*PIF3*	*SPY*	*CYCD1;1*	*CDKB2;2*	*SVP2*	*FUL2*	*FLC1*	*FLC2*
ABF1	1																			
ABI8	0.772*	1																		
BZIP12	0.673*	0.459	1																	
CYP707A1	-0.118	0.498	0.158	1																
GAI	0.808*	0.928**	0.184	0.244	1															
NCED1	0.336	0.802**	0.36	0.707*	0.712*	1														
PP2C5	-0.584	-0.069	-0.671*	0.611	-0.041	0.236	1													
PYL8	0.436	0.165	0.863**	0.28	-0.003	0.257	-0.467	1												
RGA	0.173	0.044	0.808**	0.272	-0.256	0.29	-0.397	0.969**	1											
EXPA1	-0.471	0.125	-0.223	0.886**	-0.1	0.486	0.817**	0.029	0.046	1										
GA20ox2	0.16	-0.035	-0.232	-0.393	-0.1	-0.287	-0.116	-0.512	-0.700*	-0.333	1									
GA2ox2	-0.243	0.288	-0.251	0.690*	0.345	0.647*	0.827**	-0.142	-0.09	0.795**	-0.251	1								
PIF3	-0.251	0.414	-0.112	0.875**	0.231	0.733*	0.747*	0.121	0.152	0.952**	-0.48	0.753*	1							
SPY	0.378	0.647*	0.025	0.773**	0.51	0.860**	0.453	-0.165	-0.077	0.432	0.063	0.727*	0.584	1						
CYCD1;1	-0.591	-0.484	-0.888**	0.068	-0.384	-0.42	0.734*	-0.675	-0.702*	0.458	0.321	0.285	0.371	-0.07	1					
CDKB2;2	-0.591	-0.494	-0.919**	-0.136	-0.281	-0.456	0.608	-0.786*	-0.824**	0.217	0.392	0.142	-0.003	-0.165	0.966**	1				
SVP2	0.778*	0.28	0.671*	-0.283	0.061	-0.03	-0.765*	0.156	0.077	-0.531	0.594	-0.479	-0.591	0.032	-0.628	-0.296	1			
FUL2	0.012	-0.292	0.608	-0.231	-0.38	-0.187	-0.717*	0.808**	0.779*	-0.338	-0.267	-0.597	-0.341	-0.513	-0.532	-0.447	0.415	1		
FLC1	-0.715*	-0.687*	-0.704*	-0.279	-0.368	-0.594	0.327	-0.37	-0.265	-0.033	-0.258	-0.082	-0.036	-0.479	0.724*	0.55	-0.699*	-0.208	1	
FLC2	-0.67	-0.845**	-0.562	-0.514	-0.744*	-0.804**	0.082	-0.227	-0.187	-0.227	-0.13	-0.366	-0.394	-0.769*	0.626	0.547	-0.484	0.116	0.928**	1

Correlation was analyzed using a Pearson's two-tailed test.. Significant at *$P < 0.05$, **$P < 0.01$.Gene full names are shown in Supplementary Table S1.
